# Gratitude to the Hospitals

**Published:** 1897-07-03

**Authors:** 


### Gratitude to the Hospitals.

### TWO INSTANCES AND A SUGGESTION.

Hospital Sunday affords grateful patients a seasonable opportunity of letting their gratitude take a practical shape, and we are glad to record two instances which have been brought to our notice, one that of a lady who was greatly touched by the appeal made by the Rev. Mr. Neligan at St. Stephen's, Westbourne Park, and who, in sending him an extra gift for the hospitals, writes: "It is not a year since I was placed by a friend in Charing Cross Hospital, where I underwent a serious operation for cancer: but under God's providence, a skilful surgeon, and kind nurses, my life was spared." The second instance is even more gratifying still. Mr. R. A. Owthwaite, who is one of the most zealous and best of hospital workers, received the following anonymous letter one day last week, as secretary to the City of London Lying-in Hospital:—

"London, June 22nd, 1897.

"Dear Sir,—I have much pleasure in enclosing you P.O. for 10s. 6d. as a subscription towards the City of London Lying In Hospital, and hope to continue same as long as I dwell here below.

"I admit that it has been through the want of a little consideration for others on my part that I have not contributed before, and I think that it is from the same want that thousands and tens of thousands in this country do not contribute. If only they could be brought to think that it simply requires the giving up of some small trifle (say one cigar less a week, one ribbon less, one drink less), for to become an annual subscriber to any hospital or other charitable institution of half-a-guinea means less than one halfpenny per day, twopence-halfpenny per week. It is little drops of water and sand that make the mighty ocean and the beauteous land, so that you would not mind, nor the secretary of any other hospital or institution, if all contributions came in in half-guineas, so long as they were kept free from debt. The Press has set a noble example in showing what can be accomplished with small contributions.

"I believe that were the public to be awakened in this matter there would be a very large increase of half-a-guinea subscribers.—Yours faithfully,

"Half-a-Guinea Subscriber."

We have no doubt that "Half-a-Guinea Subscriber" is correct in the view he takes that the difficulty is to get at the smaller people, and to make it easy for them to contribute their money, without deduction, to the hospitals. This difficulty, it is hoped, will be overcome by the Hospital Stamps, but they have yet to be understood by the people, and we are glad to recognise the cordial way in which hospital secretaries and managers have appreciated this fact, and are co-operating with the Prince of Wales's Fund to make them known to the class of people who can only afford small subscriptions. We should like to see the Hospital Saturday Funds all over the country take up the Prince of Wales's Hospital Stamps, and, by introducing them to the workshops, enable the artisans as a class to obtain an interesting souvenir of the Queen's Commemoration, and at the same time help the individual working men to make and keep a resolution to subscribe a small sum annually to the hospitals. They may object that the money derived from the sale of the stamps will go to the London hospitals. Our answer is that the benefits which residents in the provinces derive indirectly from the London hospitals are so great as to make the adoption of this proposal a pleasant recognition of the indebtedness which we all ought to feel to those great centres of medical and surgical progress.

